# Recurrent Ganglion Cyst In Peroneus Longus

**DOI:** 10.7759/cureus.7972

**Published:** 2020-05-05

**Authors:** Deepak Kumar, Praveen Sodavarapu, Amit K Salaria, Sharif Dudekula, Aditya Guduru

**Affiliations:** 1 Orthopaedics, Post Graduate Institute of Medical Education and Research, Chandigarh, IND; 2 Orthopaedics, All India Institute of Medical Sciences, New Delhi, IND

**Keywords:** ganglion cyst, peroneus longus, cystic lesion

## Abstract

Ganglion cysts are benign cystic lesions that are lined by a synovium and are filled with a gelatinous mucoid material. Ganglion cysts are most commonly located in the hand and the wrist. We present a rare case report of a 45-year-old male with a recurrent intramuscular ganglion cyst in the peroneus longus for two years. The patient underwent drainage one year back, but the swelling recurred one month after surgery. Magnetic resonance imaging showed a delineated, round, lobulated fluid collection consistent with the appearance of a ganglion cyst that was present within the proximal part of peroneus longus. Surgical exploration revealed an encapsulated mass present within the peroneus longus muscle belly. The complete excision of the ganglion cyst was performed, and the diagnosis was confirmed by histology. Postoperatively, at a two-month and six-month follow-up, he was completely asymptomatic with no recurrence and a normal neurological function. Ganglion, which arises from the peroneus longus muscle or tendon, presents with swelling over the lateral aspect of leg due to compression of the common peroneal nerve. Careful preservation of the nerve with complete ganglion excision gives excellent results.

## Introduction

Ganglion cysts are benign cystic lesions that are lined by a synovium and are filled with a gelatinous mucoid material. They likely result from repetitive microtrauma, mostly in areas under constant mechanical stress leading to mucinous degeneration of connective tissue, although the exact cause is doubtful. Ganglion cysts usually arise from tendon sheath, muscle, nerve, and periosteum. They are most commonly located in hand and the wrist, followed by the ankle and the foot [1]. However, the presence of an intramuscular ganglion cyst in the peroneus longus is uncommon. Ganglion cysts arising from peroneus longus muscle or tendon usually present with swelling over the lateral aspect of the leg but may cause sensory symptoms due to compression of the peroneal nerve [2]. We present a rare case report of a 45-year-old male with a recurrent intramuscular ganglion cyst in the peroneus longus.

## Case presentation

**Figure 1:**
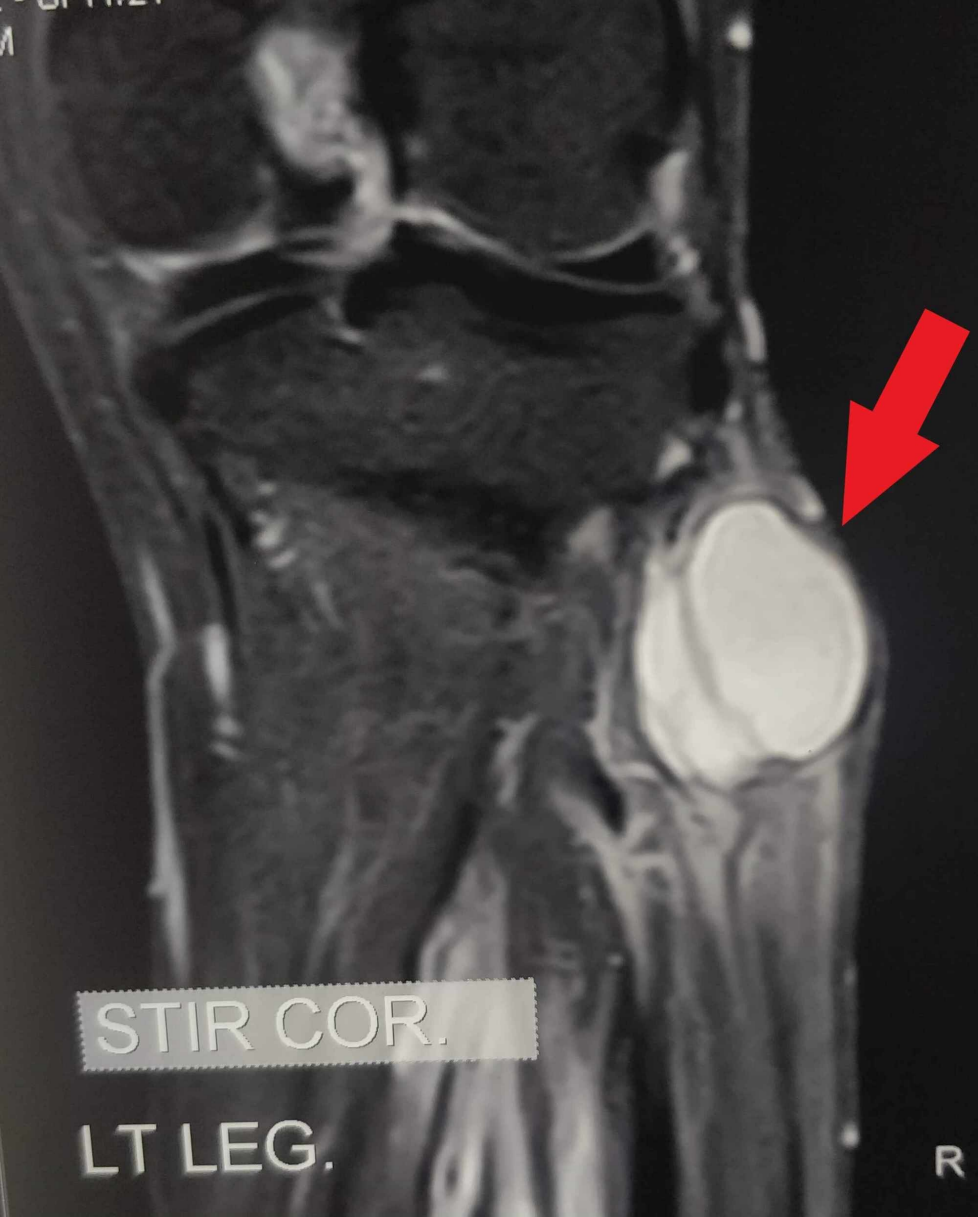
Coronal cut of magnetic resonance imaging showing ganglion cyst (pointed by the red arrow)

**Figure 2:**
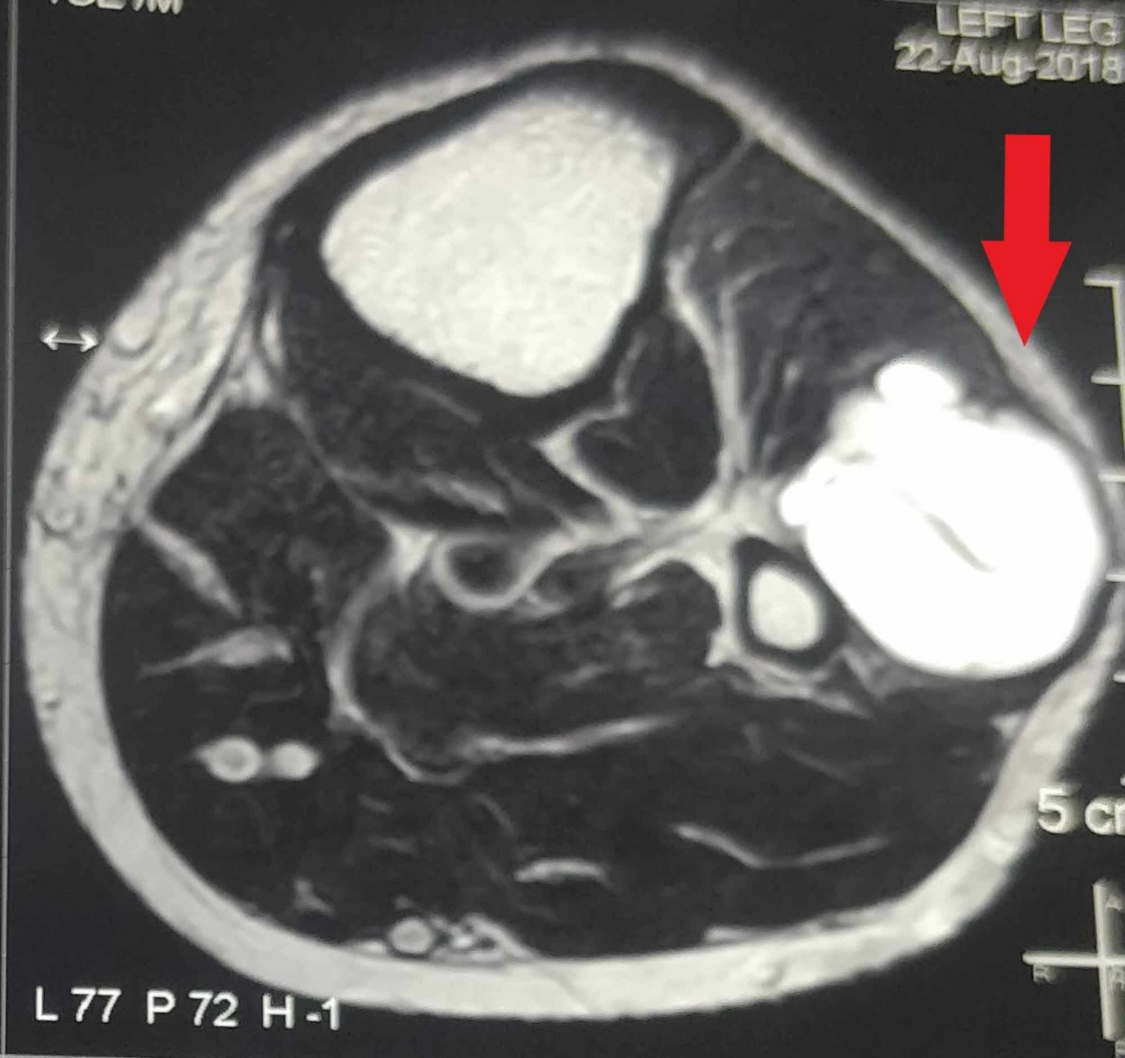
Axial cut of magnetic resonance imaging showing ganglion cyst (pointed by the red arrow)

A 45-year-old male crane operator presented with a spontaneous painless mass in the left leg for two years. Initially, he had no pain, but later, he complained of pain on prolonged standing. The swelling gradually increased in size with progressive discomfort. The patient underwent only incision and drainage of gelatinous material from the swelling without any excision one year back, but the swelling recurred one month after surgery. On examination, there was a firm, non-fluctuant, non-tender mass of size 3 cm x 7 cm in the upper third of the left leg in the proximal aspect of the lateral compartment of the leg. The patient did not have any neurological symptoms. The patient walked without a limp, and his knee had a full range of motion. The radiographic examination did not show any obvious findings. Magnetic resonance imaging was performed and reviewed by a musculoskeletal radiologist. It showed a delineated, round, lobulated fluid collection consistent with the appearance of a ganglion cyst that was present within the proximal part of peroneus longus (Figures 1, 2).

**Figure 3:**
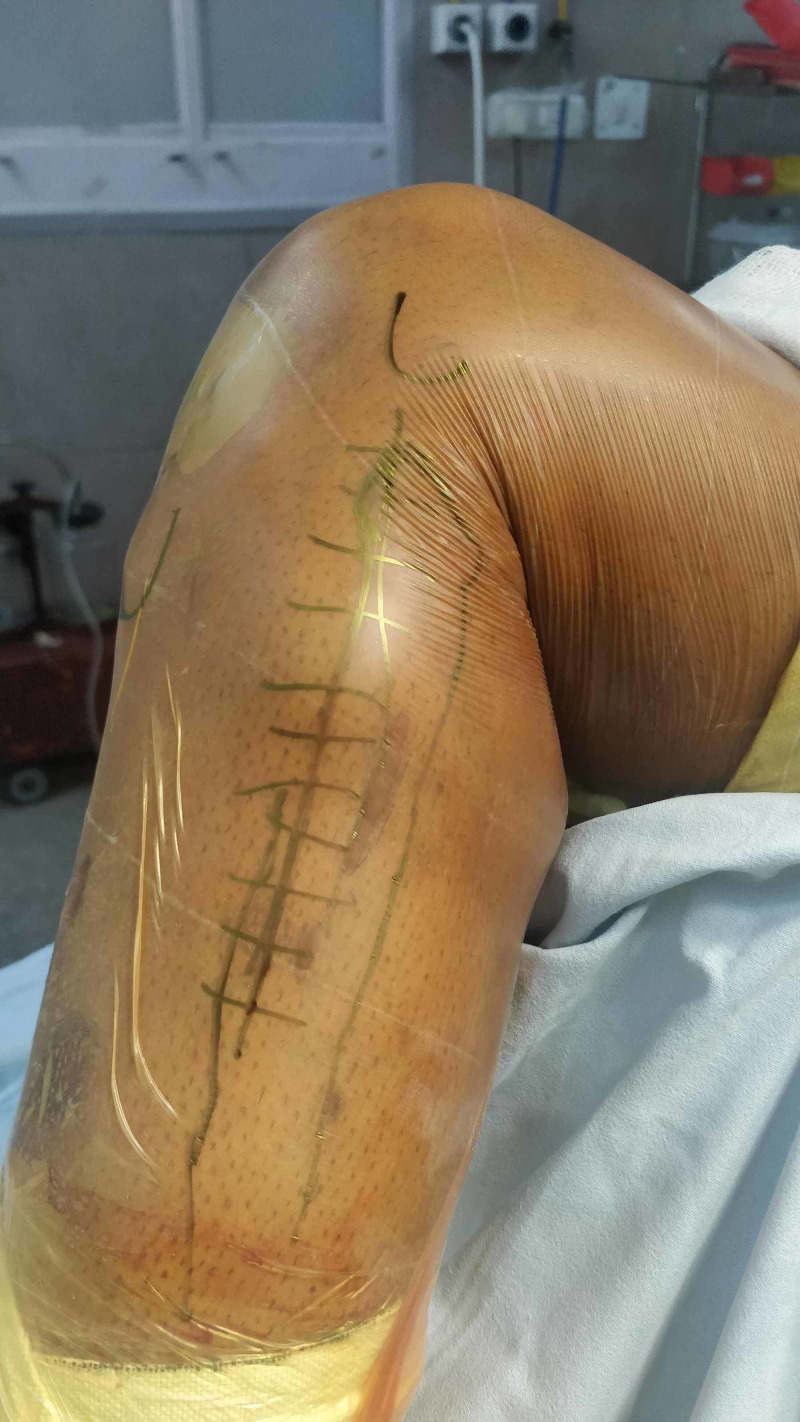
Skin marking showing fibula and line of incision

**Figure 4:**
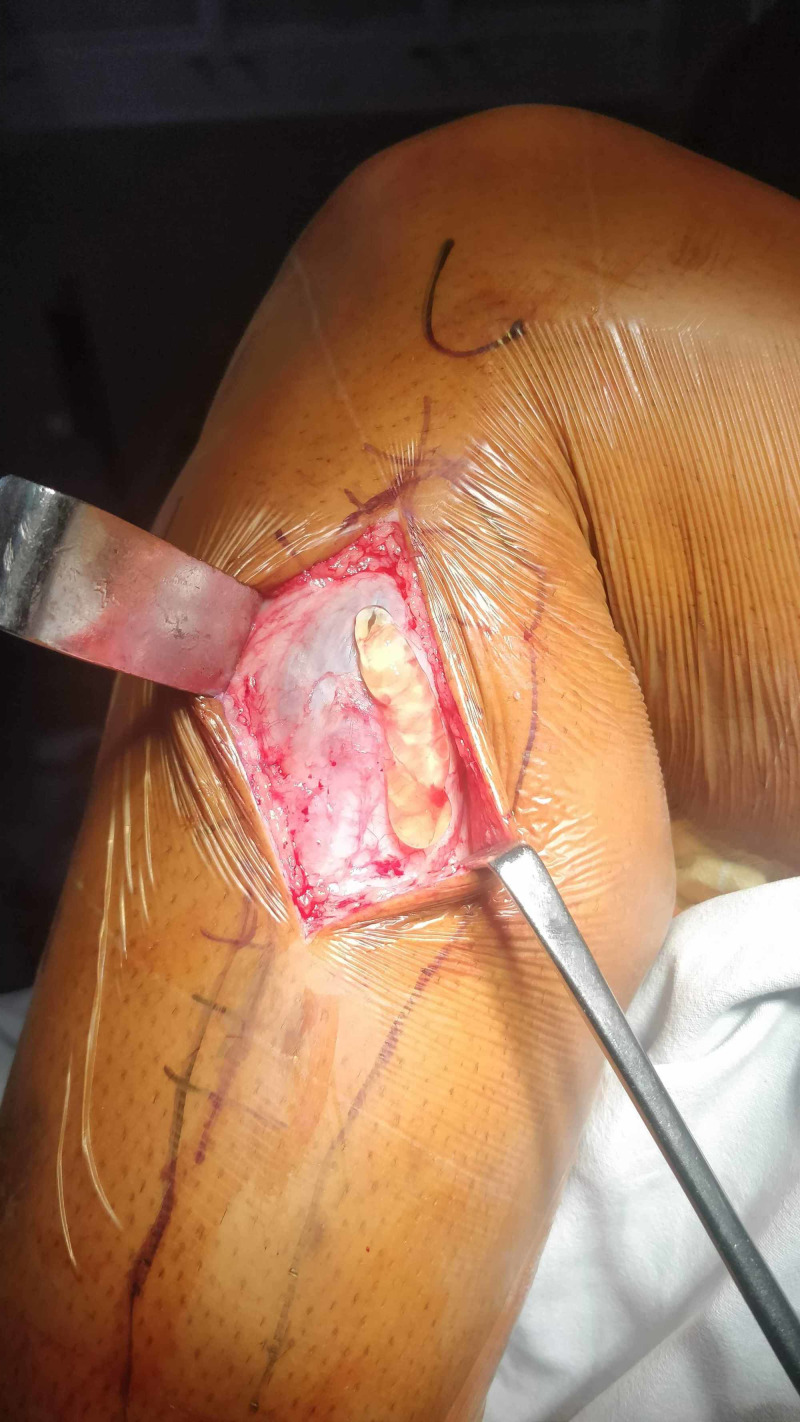
Gelatinous material of the ganglion cyst

The surgical exploration involved a longitudinal incision over the mass and revealed an encapsulated mass present within the peroneus longus muscle belly. After further dissection, the gelatinous material of an intramuscular ganglion cyst was found (Figures 3, 4)

**Figure 5:**
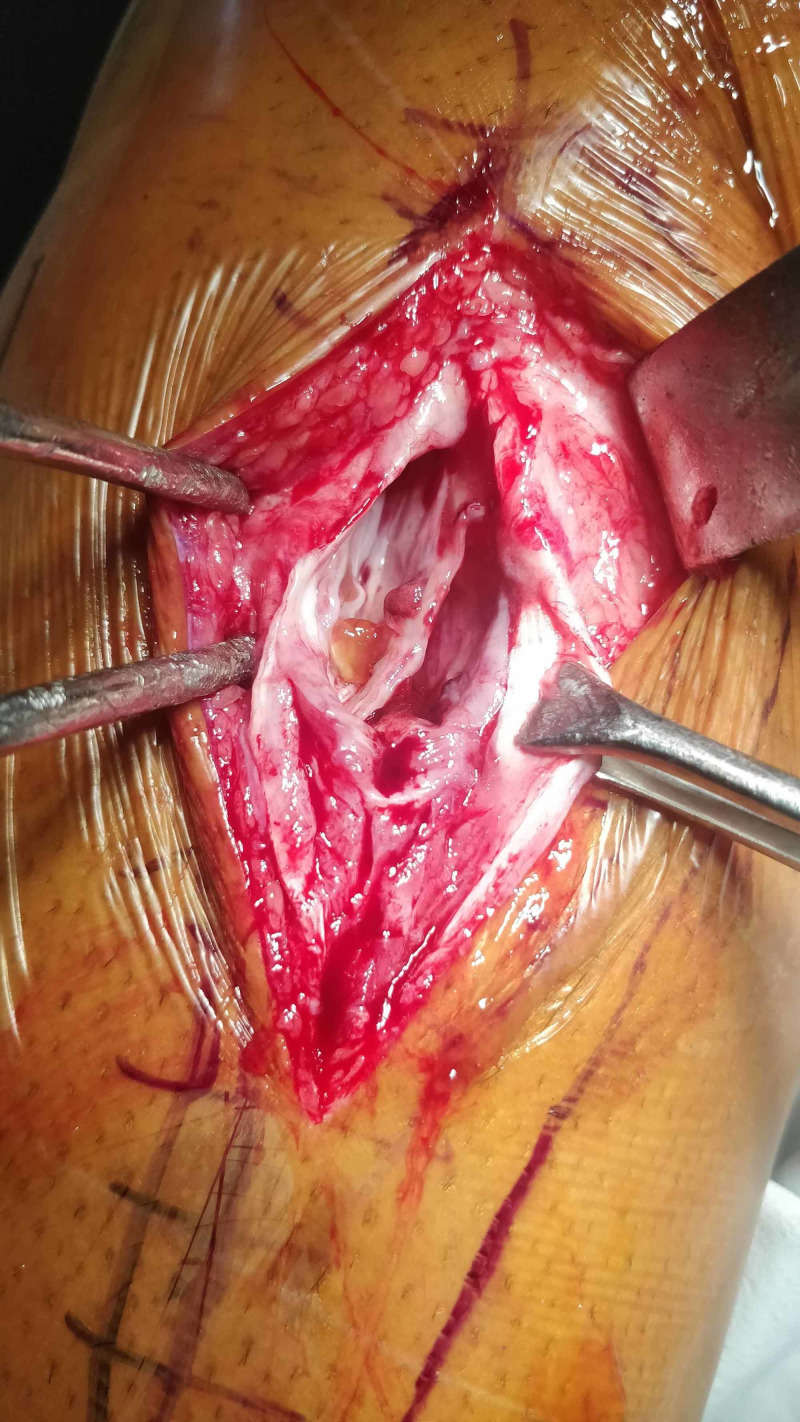
Evacuation of the gelatinous material showing the inside of the ganglion cyst

**Figure 6:**
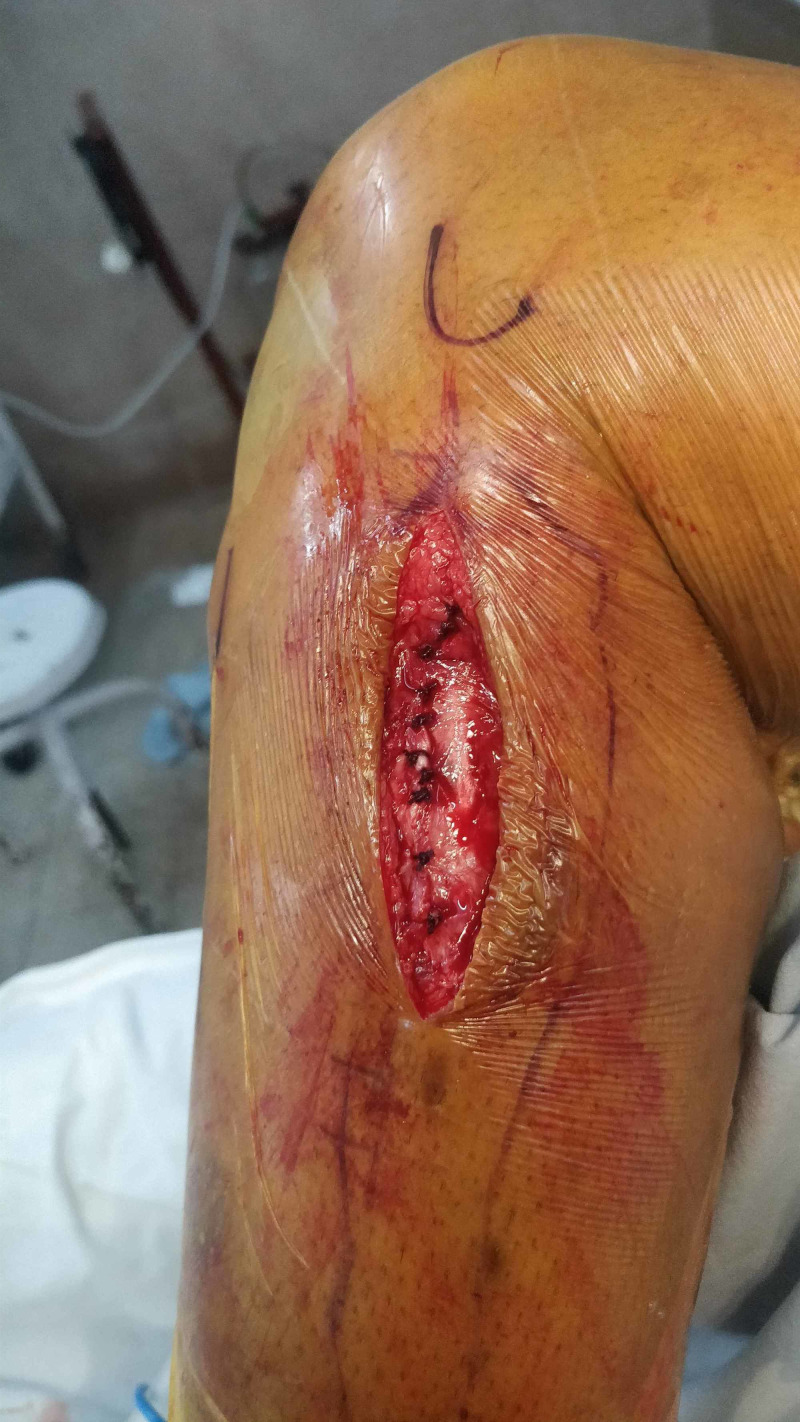
Closure of the wound in layers

No communication with the proximal tibiofibular joint was identified. Complete excision of the ganglion cyst was performed, and the investing fascia was tightly closed to prevent muscle herniation (Figures 5, 6).

**Figure 7:**
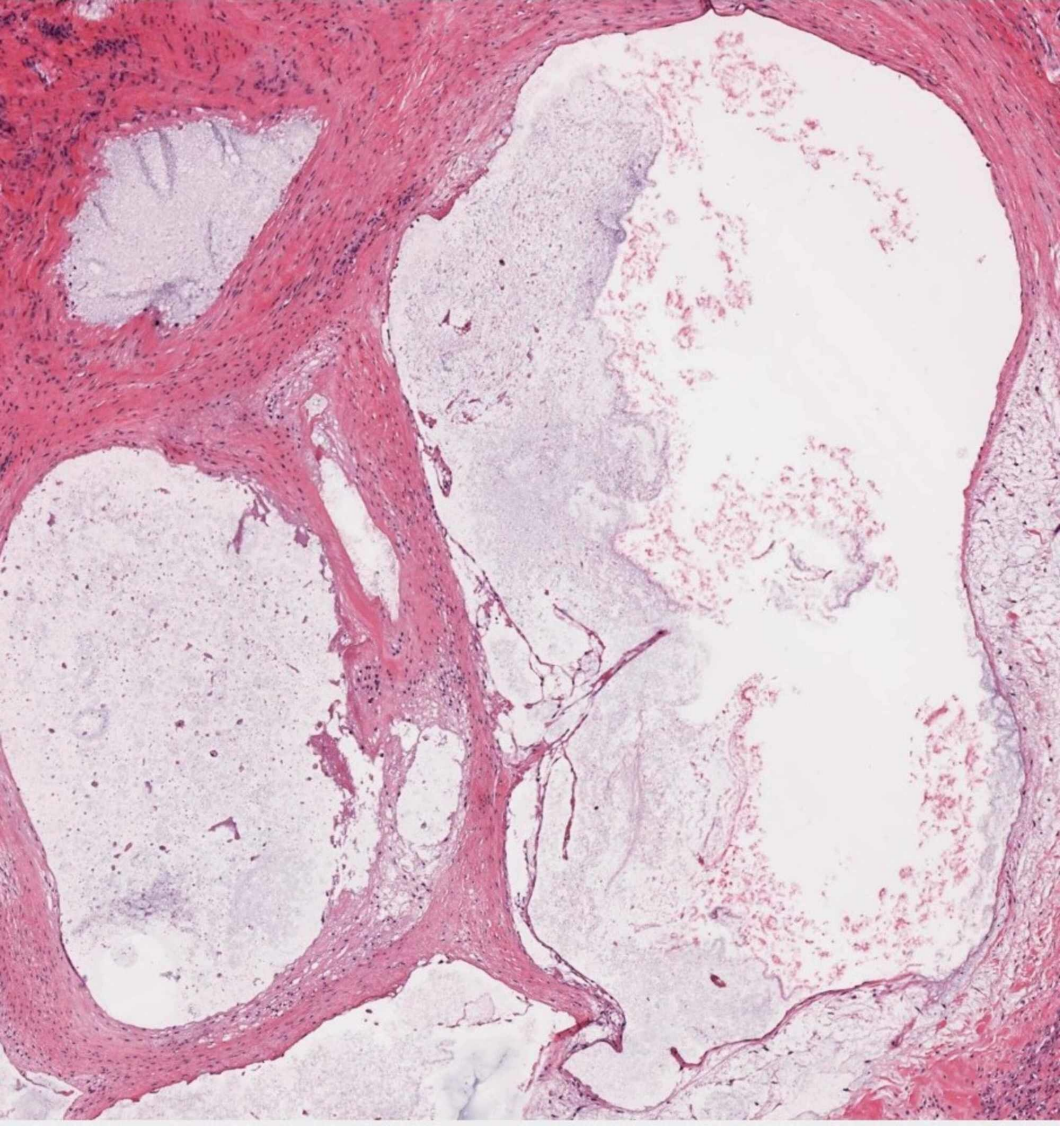
Histological picture showing multiple cystic areas with mucinous content

The diagnosis was confirmed by histology which showed multiple cystic areas with mucinous material (Figure 7).

Postoperatively at a two-month, six-month, and one-year follow-up, he was completely asymptomatic with no recurrence and a normal neurological function.

## Discussion

Ganglion cysts usually arise from joints or tendon sheath and are common around the hand and wrist but not common in the leg. Most of the ganglion cysts are asymptomatic, but they may cause pain, tenderness, or weakness in a few patients and can be a source of a cosmetic problem. Few references in literature are available with respect to the occurrence of ganglion cyst within the peroneus longus muscle. Graves et al., Muckart et al., and Marano et al. reported ganglion cysts within peroneus longus muscle, and Bowker et al. reported a case of complete replacement of peroneus longus by a ganglion [2-5].

Ganglia, which arises from the peroneus longus muscle or tendon, often present with swelling over the lateral aspect of the leg. These may cause pain, paraesthesia, and weakness in the dorsiflexion of the foot due to compression of the common peroneal nerve [3]. Such ganglion cysts are typically treated with surgical excision. Surgical management of ganglia arising from the peroneus longus involves complete excision with careful preservation of the common peroneal nerve. Alternatives to surgery include injection of sclerosing agents following aspiration of the gelatinous contents of the ganglion, as well as radiotherapy. Overall, ganglion cysts of the lower extremity have been reported to recur in approximately 10% of the cases [1].

In our case, the patient did not have any neurological symptoms and has recurred because only incision and drainage was performed without excision. Complete excision has to be done with excision of the stalk and its base in the superior tibiofibular joint if communicating with the joint to lessen the risk of recurrence.

## Conclusions

Ganglia arising from the peroneus longus muscle can be symptomatic, and surgical excision is the preferred treatment. Complete excision of the ganglion along with its stalk and base must be done to decrease the risk of recurrence. Careful preservation of the nerve with complete ganglion excision gives excellent results.
